# Parkinson’s Disease in Saudi Patients: A Genetic Study

**DOI:** 10.1371/journal.pone.0135950

**Published:** 2015-08-14

**Authors:** Bashayer R. Al-Mubarak, Saeed A. Bohlega, Thamer S. Alkhairallah, Amna I. Magrashi, Maha I. AlTurki, Dania S. Khalil, Basma S. AlAbdulaziz, Hussam Abou Al-Shaar, Abeer E. Mustafa, Eman A. Alyemni, Bashayer A. Alsaffar, Asma I. Tahir, Nada A. Al Tassan

**Affiliations:** 1 Behavioral Genetics unit, Department of Genetics, King Faisal Specialist Hospital and Research Center, Riyadh, Saudi Arabia; 2 Department of Neurosciences, King Faisal Specialist Hospital and Research Center, Riyadh, Saudi Arabia; 3 King Abdulaziz City for Science and Technology, Kingdom of Saudi Arabia, Riyadh, Saudi Arabia; National Institutes of Health, UNITED STATES

## Abstract

Parkinson’s disease (PD) is one of the major causes of parkinsonism syndrome. Its characteristic motor symptoms are attributable to dopaminergic neurons loss in the midbrain. Genetic advances have highlighted underlying molecular mechanisms and provided clues to potential therapies. However, most of the studies focusing on the genetic component of PD have been performed on American, European and Asian populations, whereas Arab populations (excluding North African Arabs), particularly Saudis remain to be explored. Here we investigated the genetic causes of PD in Saudis by recruiting 98 PD-cases (sporadic and familial) and screening them for potential pathogenic mutations in PD-established genes; *SNCA*, *PARKIN*, *PINK1*, *PARK7*/*DJ1*, *LRRK2* and other PD-associated genes using direct sequencing. To our surprise, the screening revealed only three pathogenic point mutations; two in *PINK1* and one in *PARKIN*. In addition to mutational analysis, CNV and cDNA analysis was performed on a subset of patients. Exon/intron dosage alterations in *PARKIN* were detected and confirmed in 2 cases. Our study suggests that mutations in the ORF of the screened genes are not a common cause of PD in Saudi population; however, these findings by no means exclude the possibility that other genetic events such as gene expression/dosage alteration may be more common nor does it eliminate the possibility of the involvement of novel genes.

## Introduction

Parkinson's disease (PD) is a movement disorder that was first described in 1817 [1], with a prevalence of approximately 1–2% at age 60 [2]. It is characterized by the occurrence of four cardinal symptoms: bradykinesia, resting tremor, rigidity and postural imbalance. These motor manifestations are attributable to dopamine deficiency in the striatum due to degeneration of the dopaminergic neurons within the substantia nigra pars compacta (SNpc).

The past two decades have witnessed rapidly emerging evidence for the key role of genes in the etiology of Parkinson’s disease (PD), supplanting a long-held view about the non-genetic nature of the disease. Intensive research, following the discovery of α-Synuclein (*SNCA* [MIM 163890]), has hitherto identified more than 16 PD related loci [3]. Despite the recent advances, only 10% of the familial cases and less than 5% of the sporadic ones can be ascribed to monogenic mutations in either autosomal recessive (*PARKIN* [MIM 602544], *PINK1* [MIM 608309] and *PARK7*/*DJ1* [MIM 602533]) or autosomal dominant (*SNCA* and *LRRK2* [MIM 609007]) genes [4–6]. However, the phenotypic commonalities in familial and sporadic PD has led researchers to believe that both forms of the disease may share some mutual pathways. Moreover, PD is expected to impose a major socioeconomic burden on aging populations. One way to relieve this burden is by gaining clearer understanding of the genetic etiology of the disease that may aid in designing effective diagnostic and therapeutic strategies. In this study we sought to determine the genetic causes of PD in Saudi patients. Such studies are lacking with the exception of a single report of a missense mutation in *PINK1* in an extended Saudi family with Early-onset PD [7].

## Subjects and Methods

### Subjects

A total of 98 individuals with PD, of which 33 were familial [24 autosomal recessive (AR) and 9 autosomal dominant (AD)], 63 were sporadic and 2 cases with incomplete family history data, were enrolled in this study. This study was approved by the Institutional Review Board of King Faisal specialist hospital and Research Center (project RAC# 2110035). Approved written consent forms were obtained from all subjects prior to their enrollment. Neurological assessment of patients was performed by movement disorder specialists and diagnosis of PD was established according to the accepted criteria. Patients were grouped as familial (with at least one reportedly affected first- or second-degree relative) or sporadic (no family history of the disease), and as Juvenile onset (JO; age of onset (0–20) years), Early onset (EO; (20–50) years) and late onset (LO; >50 years). Demographic and clinical features of patients are summarized in Table 1. Detailed clinical features of selected familial and sporadic cases are described in S1 File.

**Table 1:** Summary of demographic and clinical features of subjects with PD.

	Form of PD	SP	FM	NR
Age at onset	**Number**	63	33	2
	**Gender (M/F)**	48/15	26/7	2/0
	**JPD**	3	5	0
	**YOPD**	31	18	1
	**LOPD**	24	6	0
	**NR**	5	4	1
	**Asymmetry (Y/N/NR)**	(26/12/25)	(16/2/14)	(0/1/1)
	**Tremors (Y/N/NR)**	(43/7/13)	(21/5/7)	(0/1/1)
	**Bradykinesia (Y/N/NR)**	(49/1/13)	(25/0/8)	(1/0/1)
Clinical features	**Rigidity (Y/N/NR)**	(46/1/16)	(24/1/8)	(1/0/1)
	**Dystonia (Y/N/NR)**	(16/32/15)	(12/14/7)	(1/0/1)
	**Gait impairment (Y/N/NR)**	(24/24/15)	(13/12/8)	(0/1/1)
	**Depression (Y/N/NR)**	(22/26/15)	(10/15/8)	(1/0/1)
	**Hallucination (Y/N/NR)**	(12/36/15)	(6/17/10)	(1/0/1)
	**Dementia (Y/N/NR)**	(7/40/16)	(3/23/7)	(0/1/1)
	**Pyramidal signs (Y/N/NR)**	(2/46/15)	(0/26/7)	(0/1/1)
	**Ataxia (Y/N/NR)**	(2/45/16)	(2/23/8)	(0/1/1)
	**LDOPA response (Y/N/NR)**	(46/3/14)	(23/2/8)	(1/0/1)
	**Wearing off (Y/N/NR)**	(42/6/15)	(214/8)	(0/1/1)
	**Peak dose Dyskinesia (Y/N/NR)**	(32/15/16)	(16/7/10)	(0/1/1)

Key: Sporadic, SP; Familial, FM; Male, M; Female, F; Juvenile onset PD, JOPD; Early onset PD, EOPD; Late onset PD; LOPD, Not reported, NR; Yes, Y; No, N.

### Experimental procedures

#### Mutational analysis of PD genes

Peripheral blood specimens were collected from patients for genomic DNA isolation using standard protocols. The entire coding sequence, including intron/exon boundaries, for common PD-genes; *SNCA*, *PARKIN*, *PINK1*, *PARK7/DJ1*, *LRRK2* and other PD-associated genes including; *UCHL1*[MIM 191342], *GIGYF2*[MIM 612003], *FBXO7* [MIM 605648], and *VPS35* [MIM 601501] was investigated in patients by means of direct sequencing using ABI Prism Big Dye Terminator ready reaction cycle sequencing kit (Applied Biosystems, Foster City, CA, USA). All 98 DNA samples were sequenced for the common PD-genes (mentioned above), while 82 out of 98 were sequenced for both common and other PD-associated genes (see above). Primers and PCR conditions are available upon request. Novel non-synonymous sequence variants with pathogenic prediction were screened in 700 Saudi normal controls, whereas those with benign predicted effect were screened in around 100 ethnically matching healthy controls.

#### RT–PCR

Total RNA was extracted from lymphocytes using PAXgene Blood RNA Kit (PreAnalytiX GmbH, Switzerland), followed by cDNA synthesis using Reverse Transcription System (Promega, CA, USA). Direct PCR amplification of *LRRK2*, *SCNA*, *PINK1*, *PARKIN* and *PARK7*/*DJ1* cDNA was performed using gene-specific primers and β-actin was used as an internal control. The resulting amplicons were evaluated by electrophoresis on 2% Agarose gel. For primers sequences, PCR products size and transcripts information see S2 Table. Representative bands were sequenced to confirm origin.

#### Detection of copy number alterations

Out of the 98 samples, 25 representative samples were screened for dosage alterations of both common and associated-PD genes using the Cyto Scan HD array (Affymetrix, Santa Clara, CA,USA) which contains 2.6 million markers for genome coverage. The data was analyzed using the Chromosome Analysis Suite version Cyto 3.0 using GRC 38/hg19 of the UCSC Genome Browser. A threshold of log2 ratios of more than 0.58 for CNV gains and less than -1 for CNV losses was used.

### In silico analysis

The disease-causing potential of the detected novel non-synonymous variants was assessed using 4 prediction tools namely, MutationTaster (http://www.mutationtaster.org/), SIFT, PROVEAN and PolyPhen2 (http://genetics.bwh.harvard.edu/pph2/). Classification of the previously reported non-synonymous variants was in accordance with The Human Gene Mutation Database (HGMD; http://www.hgmd.cf.ac.uk/ac/index.php) terminology. Wild-type and mutant PINK1 3D-models were predicted using protein structure prediction software I-TASSER [8]. Various protein analytical tools, were utilized to choose the best model; including PROSA [9], RAMPAGE [10] and NIH-SAVES server (http://services.mbi.ucla.edu/SAVES/) of which ERRAT was performed [11]. PyMol (Molecular Graphics System, Version 1.2r3pre, Schrödinger, LLC) was used for protein superimposition and molecular graphics. PMUT [12] and I-Mutant [13] were used to predict protein pathological character and stability upon single point mutation, respectively. As for PARKIN, wild-type and mutant 3D-models were generated in the same manner, except that homology-based protein structure prediction software (MODELLER; https://salilab.org/modeller/) [14] was used instead of I-TASSER.

## Results

In the present study we detected; a total of 118 different sequence variants including three pathogenic point mutations and exon/intron dosage alteration of *PARKIN*. Of the detected sequence variants, 9 were HGMD-listed non-synonymous, 91 were reported in dbSNP (S1 Table) and 18 were novel variants (Table 2).

**Table 2:** Novel sequence variants detected in this study.

	Variant		Prediction tools						
Gene/PCR primer/position	cDNA	Protein	MutationTaster	PolyPhen-2	SIFT	PROVEAN	Het/Hom	FM/SP	*Frequency
*PARKIN*/Exon4	c.532C>T	Q178X	disease causing	n.a.	n.a.	n.a.	0/1	0/1	n.a.
*PARKIN*/Exon5	c.583G>C	E195Q	polymorphism	benign	tolerated	Neutral	1/0	0/1	0/96
*PARKIN*/Exon6	c.718A>G	T240A	polymorphism	benign	tolerated	Neutral	2/0	2/0	0/96
*PINK1*/Exon2	c.565G>A	G189R	polymorphism	benign	tolerated	Neutral	1/0	0/1	2/96
*PINK1*/Exon6	c.1225G>A	G409R	disease causing	probably damaging	deleterious	damaging	0/2	2/0	0/1000+
*PARKIN*/Exon12/3'UTR	c*61C>T	_	_	_	_	_	1/0	0/1	n.a.
*PINK1*/Exon3/intron3	c.776+22G>A	_	_	_	_	_	3/0	0/3	n.a.
*PINK1*/Exon5	c.966C>T	P322P	_	_	_	_	12/0	4/8	n.a.
*PINK1*/Exon6	c.1237C>T	L413L	_	_	_	_	21/0	6/15	n.a.
*LRRK2*/Exon2/intron1	c.152-14C>T	_	_	_	_	_	5/0	0/5	n.a.
*LRRK2*/Exon11/intron11	c.1288+164T>G	_	_	_	_	_	1/0	0/1	n.a.
*LRRK2*/Exon44/intron43	c.6381-19T>G	_	_	_	_	_	1/0	1/0	n.a.
*LRRK2*/Exon47/intron46	c.6844-18_6844-17insT	_	_	_	_	_	0/3	1/2	n.a.
*GIGYF2*/Exon6/intron5	c.42-57T>A	_	_	_	_	_	4/1	0/5	n.a.
*GIGYF2*/Exon30	c.3753A>G	Q1251Q	_	_	_	_	1/0	0/1	n.a.
*FBXO7*/Exon3/intron3	c.645+78A>G	_	_	_	_	_	1/0	0/1	n.a.
*FBXO7*/Exon8/intron7	c.1145-112_1145-111insTTC	_	_	_	_	_	0/13	2/11	n.a.
*PARK7*/*DJ1*/Exon4/intron4	c.252+57G>A het	_ _	__	__	__	__	3/0	2/1	n.a.

Key: Heterozygous, Het; Homozygous, Homo; Familial, FM; Sporadic, SP; Not available, n.a.

* Frequency: No. of control carriers/ total No. of controls.

### Reported variants

Nine HGMD-listed variants were detected in our patients, of which 5 were classified as disease-causing mutations and 4 as disease-associated polymorphisms. Two of the disease-causing mutations were present in *PARKIN*; p.Q34R and p.T240M, two were present in *PINK1*; p.T313M and p.E476K and one was found in *PARK7*/*DJ1*; p.R98Q [7, 15–21]. As for the disease-associated polymorphisms: two were present in *PARKIN*; p.S167N and p.V380L, one was present in *UCHL1*; p.S18Y and another was found in *LRRK2*; p.N551K [22–26]. Genotypes and frequencies of all detected variants are described in S1 Table. All the allelic variants occurred in heterozygous state apart from two sporadic cases; one (PD-108) homozygous for p.T313M missense mutation located in *PINK1* and the other homozygous for p.S18Y substitution in *UCHL1*. In addition, two *PARKIN* variants (p.V380L and p.T240M) were found to be shared in a heterozygous state in two affected siblings (FM 19) (S1A Fig). The heterozygous p.R98Q substitution in *PARK7*/*DJ1* was detected in two affected siblings and their unaffected father (FM92) (S1C Fig) in addition to a single sporadic case. For detailed clinical features of the cases see S1 File.

### Novel variants

Eighteen novel sequence variants were detected in our samples; 10 were non-coding (9 intronic and one in the 3’-UTR) and eight were exonic. Out of the eight exonic variants, one was a nonsense mutation (p.Q178X in *PARKIN*) four were non-synonymous (p.E195Q and p.T240A in *PARKIN*; p.G189R and p.G409R in *PINK1*) and three were synonymous substitutions (p.P322P and p.L413L in *PINK1* and Q1251Q in *GIGYF2*). Genotypes and frequencies are described in Table 2.

**Fig 1:**
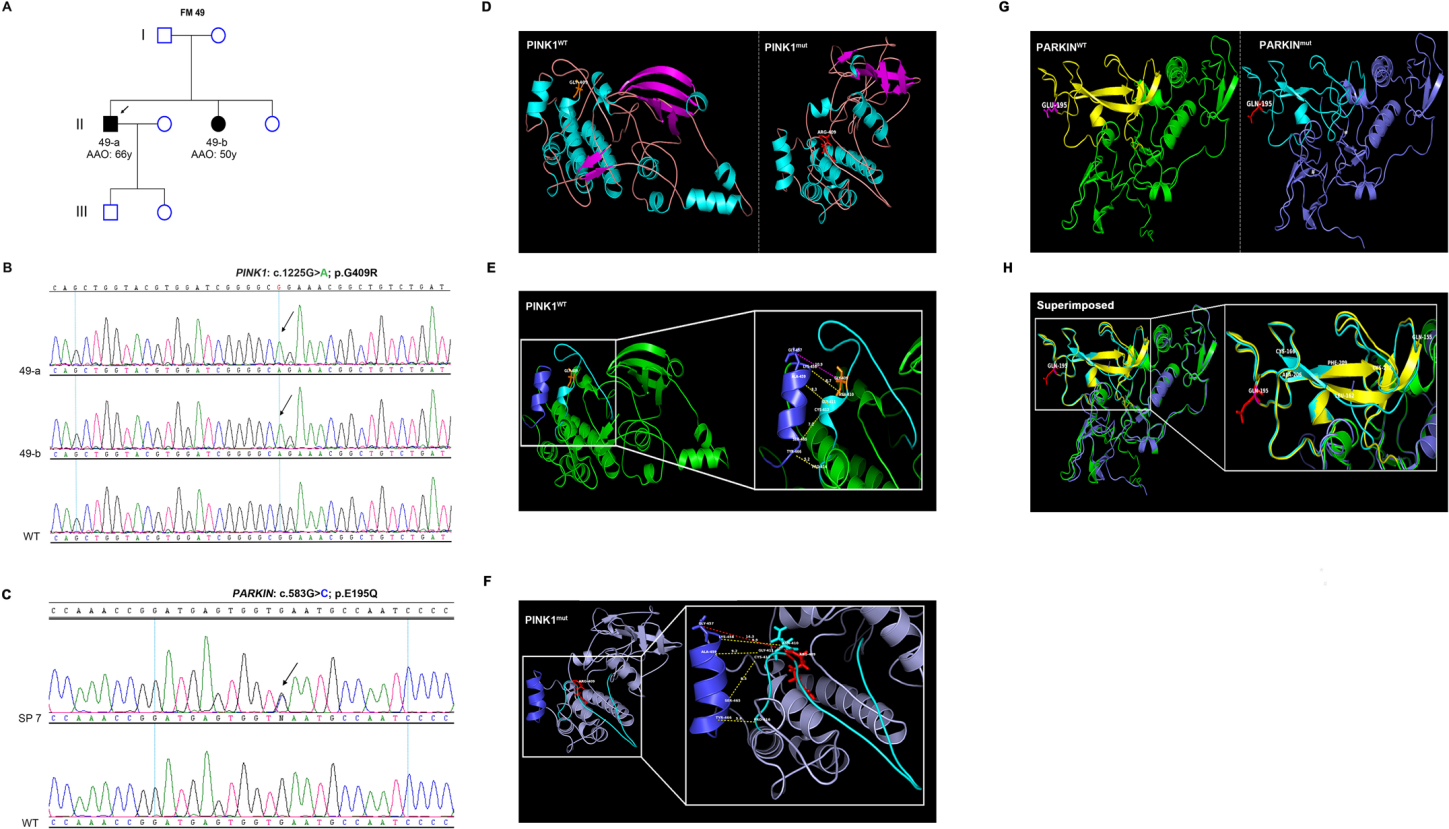
Genetic characterization of *PARKIN* (p.G409R) and *PINK1* (p.E195Q) variants and their predicted functional impact. (A) Pedigree of FM 49 with LOPD. (B) Part of the sequencing chromatogram of *PINK1* exon 6 showing homozygous c.1225G>A mutation (corresponding to p.G409R substitution) in 49-a and 49-b but not in WT. (C) Part of the sequencing chromatogram of *PARKIN* exon 5 showing heterozygous c.583G>C variant (corresponding to p.E195Q substitution) in SP-7. (D) Ribbon presentation of PINK1^WT^ and PINK1^mut^ structural models. The secondary structures are colored as follows: β-strands (magenta), α-helices (cyan), coils (light pink). (E) PINK1^WT^. (F) PINK1^mut^. The spatial distance between the P+1 binding motif (cyan) and helix G (blue), measured in Angstrom (Å), is increased in PINK1^mut^ compared to PINK1^WT^. A close-up view of the activation loop (aa 384–417) containing the P+1 binding motif and the helix G is represented [27, 28]. (G and H) p.E195Q has a very subtle impact on the protein conformation. Ribbon presentation of PARKIN^WT^ and PARKIN^mut^ structural models. The UPD, is shown in (yellow) or (cyan) in PARKIN^WT^ and PARKIN^mut^, respectively. (G) Positions of the missing β-strand and α-helix are indicated by the asterisk and the hash symbols, respectively. (H) Superimposition of PARKIN^WT^ and PARKIN^mut^ showing parts of the protein (indicated by a hash symbol) that had lost the β-strand structure and adopted a random coil instead. Age at onset: AAO. Years: y. WT: wild-type.

The novel p.G409R substitution in *PINK1* was identified in a homozygous state in two affected siblings form an AR family (FM 49) (Fig 1A and 1B) (for detailed clinical features see S1 File) and was absent in more than 700 Saudi normal controls. While p.G189R substitution was identified in one sporadic case and two controls (Table 2).

As for *PARKIN* novel variants, the nonsense mutation (p.Q178X) occurred in a homozygous state in a single sporadic case (PD-110) with EOPD (S6 Fig), while the two non-synonymous substitutions (p.E195Q and p.T240A) were found to be shared (in a heterozygous state) with another variant either in a different gene or within *PARKIN* itself. For instance, p.E195Q substitution was found to be shared with p.N551K polymorphism in *LRRK2* in a single sporadic case (SP 7) (S1D Fig). Similarly, p.T240A novel variant was found to be shared with the previously reported *PARKIN* mutation (p.Q34R) in a single proband and his affected father (FM 6) (S1B Fig). While p.G189R was found in a heterozygous state in one sporadic case and two controls.

Apart from *PINK1* (p.G409R) missense mutation and *PARKIN* (p.Q178X) nonsense mutation, none of the novel variants detected in this study, is likely to be pathogenic as suggested by multiple prediction tools including (MutationTaster, PolyPhen2, SIFT and PROVEAN (http://provean.jcvi.org/index.php)) (Table 2). The G^409^ residue was highly evolutionary conserved (S2A Fig) and p.G409R substitution was absent in ~700 Saudi normal controls (Table 2).

### cDNA analysis

Gene expression evaluation was performed on a subset of patients’ samples (n = 12) from which RNA was available using primers designed to amplify specific portions of *LRRK2*, *SNCA*, *PINK1*, *PARKIN* or *PARK7/DJ1* transcripts. Amplicons of the correct predicted sizes corresponding to the selected genes transcripts were successfully generated in all tested samples with the exception of one sample (PD-25a) that produced a shorter transcript of *PARKIN* (~160bp) which appears to be missing a 137bp fragment equivalent to the size of exon 7 (S3C Fig) The presence of transcripts for the selected genes in the tested PD samples, suggest that mutation(s) in the regulatory elements or deep intronic regions of these genes are more likely to be absent.

### CNV analysis

Interestingly, CNVs were observed only in *PARKIN*. Heterozygous and homozygous exonic loss and partial intronic loss was observed in 8 cases (S3 Table). Loss of exon 3 and parts of introns 2 and 3 was detected in two different families (FM 19 and FM 21). In FM 19 both affected siblings were heterozygous for the loss (S4A Fig), whereas in FM 21 only the affected father was homozygous for the loss, while his unaffected son was heterozygous for the loss (S4B Fig). Heterozygous gain of exon 6 and partial loss of introns 5 and 6 was observed in one familial case with JOPD (FM 23) (S4C Fig). Homozygous loss of exon 7 and partial loss of introns 6 and 7 were detected in two affected siblings displaying EOPD (S4D Fig). This deletion was confirmed at a cDNA level in the proband (25-a) from which RNA sample was available (S3C Fig). A CNV gain covering exon 6 was observed in a proband of FM-23. Moreover, one sporadic case (SP-103) with EOPD harbored homozygous deletions of multiple exons (3 and 4), intron 3 as well as partial loss of introns 2 and 4. Exons 3 and 4 deletions were confirmed in this patient by PCR analysis of gDNA for *PARKIN* exon 3 or 4 co-amplified with a separate gene as an internal control (S5 Fig). For detailed clinical description of the patients with exonic deletions (PD-25a and PD-103) see S1 File.

### In silico structural analysis of *PARKIN* (p.E195Q) and *PINK1* (p.G409R) novel variants

Encouraged by the type of amino acid change (from non-polar amino acid to a positively charged one) (Fig 1B), the absence in ~1400 chromosomes, the evolutionary conservation (S2A Fig) and the damaging prediction analysis of p.G409R substitution (Table 2), we decided to take advantage of computational tools to explore its impact on PINK1 function and structure. We therefore opted to model only the kinase domain (aa 162–512) harboring Gly^409^ residue (Fig 1D) (see S1 File for detailed description). The *In silico* analysis revealed loss of 4 α-helices in PINK1^mut^ that could disturb the domain conformation or stability. Furthermore, the spatial distance between the P+1 binding motif (responsible for substrate binding) and the adjacent helix G, the two segments comprising the P+1 specificity pocket [28], was increased, possibly to accommodate the large side chain of Arginine (Fig 1E and 1F).

Despite the neutral prediction analysis of p.E195Q variant (Table 2), the substitution of a negatively charged amino acid with a polar uncharged one (Fig 1C), the conservation of the native amino acid throughout mammals (S2B Fig), its absence in 192 control chromosomes, and its position within the Unique Parkin domain (UPD), a Zn^+2^ –binding domain important for substrate binding and ubiquitination [29, 30], prompted us to investigate the structural and functional consequences of this substitution. Our PARKIN structural models were based on a previous PARKIN model, spanning amino acid residues (141–465), deposited in the RCSB (http://www.rcsb.org/pdb/home/) database under the accession number (PDB-ID# 4K95) (see S1 File for detailed description). Although p.E195Q substitution caused very subtle changes in protein folding, a number of secondary structure changes have been observed (Fig 1G and 1H). These include alterations in the number and/or length of secondary structural elements compared to the predicted PARKIN ^WT^ model. Firstly, the loss of one β-strand and one α-helix (Fig 1G). Secondly, two β-strands (within the UPD domain; aa 142–227 [30]) were shortened, one by 8 amino acids and the other by 4 amino acids (Fig 1H).

## Discussion

In the current study we set out to investigate the genetic basis of PD in Saudi patients. We decided to use a more general categorization of either familial or sporadic based on the presence or absence of positive family history and subsequently screen all patients for mutations in both PD-autosomal and PD-recessive genes.

Interestingly, our sequence analysis of well-established PD-autosomal recessive (*PARKIN*, *PINK1* and *PARK7*/*DJ1*) and PD-autosomal dominant (*SCNA* and *LRRK2*) genes in families with the corresponding mode of inheritance as well as sporadic cases, detected only three pathogenic point mutations; two of which were missense [p.G409R in *PINK1* (Fig 1A and 1B) and p.T313M in *PARKIN* (S1 Table)], while the third was a nonsense mutation [p.Q178X in *PARKIN*, (S6 Fig)]. The lack of *LRRK2* mutations, a common cause of PD in North African Arabs [31, 32] and Ashkenazi Jews [33], in our AD and sporadic PD-cases suggests that PD is genetically more heterogeneous in Saudis compared to other Middle Eastern populations.

The p.G409R variant was predicted to be pathogenic by four softwares (Table 2). Our *in silico* protein modeling predicted that PINK1^mut^ lacked secondary structure elements (4 α-helices) and the substitution of Gly^409^ with Arg increased the spatial distance between P+1 binding motif and the adjacent helix G (Fig 1E and 1F). Residues in P+1 binding motif and helix G are involved in forming a P+1 specificity pocket necessary for kinase-substrate interaction [28, 34]. Potential implications of such changes in the secondary structures and the P+1 specificity pocket of PINK1 may include; compromised structural integrity of the domain and altered substrate recognition specificity, which may interfere with PINK1 normal kinase activity. This is in line with previous studies demonstrating the adverse effect of substitution with Val at the same residue on PINK1 kinase activity and substrate recognition [27, 28, 35, 36]. However, functional and cellular studies are required to confirm the predicted consequences. The other pathogenic *PINK1* mutation detected in this study, is the p.T313M substition previsiouly described in a Saudi and a Chinese kindred with early onset PD [7, 16] and was shown to cause neuronal toxicity and abnormal mitochondrial accumulation [37]. This mutation was present in a homozygous state in one patient (PD-108) with no consanguinty or positive family history reported, however, DNA from parents was not available for carrier status assessment.

As for *PARKIN* novel variants, p.Q178X truncating mutation in exon 4 had the ability to bypass nonsense-mediated mRNA decay as demonestrated by the presense of *PARKIN* transcript (S3C Fig) and therefore, may give rise to a defective protein product missing 287 amino acid residues. The second novel *PARKIN* variant is p.E195Q. *In silico* modeling suggests that this substitution has a subtle effect on protein confirmation (Fig 1G). However, alterations in the number and/or length of secondary structures were observed (Fig 1G and 1H). The analysis revealed that PARKIN ^mutt^ had lost one β-strand and one α-helix (Fig 1G). Moreover, a portion of the two central anti-parallel β-strands of the UPD Zn-binding fold, transformed to random coil structure (Fig 1H). Disruption of Zn^2+^ coordination is one possible outcome of such a structural transition especially since the altered strands contain at least one proposed Zn^2+^ coordinating residue (Cys^212^). Proper Zn^2+^ ions coordination is perquisite for the maintenance of PARKIN 3-D structure, this is supported by studies on Zn^2+^-binding domains showing that EDTA-induced- Zn^2+^ removal causes protein unfolding and therefore, would be expected to interfere with its normal function [29, 38, 39].

The other novel *PARKIN* variant (p.T240A) was detected in a single familial case in a heterozygous state (FM 6, proband and affected father) (S1B Fig). This mutation was absent in 192 control chromosomes, had neutral prediction analysis (Table 2) and was modestly conserved (S2D Fig). However, mutations at the same residue (p.T240R/M) have been speculated to alter a possible phosphorylation site for casein kinase II (CK-II) [19, 40], or to disrupt PARKIN association with Ubiquitin-conjugating enzyme (E2) necessary for ubiquitin-dependent proteasomal degradation [41–43].

The previously reported missense mutations identified in this study, excluding p.T313M in *PINK1*, (S1 Table), are less likely to be disease-causing in our patients due to one or a combinations of the following; presence in normal controls, neutral prediction analysis, reported lack of co-segregation in familial cases or lack of/equivocal evidence for functional impact [20, 44–48]. Although a heterozygous variant occurring in autosomal recessive gene is unlikely to be sufficient to cause the disease by itself, it may, however, confer risk in conjunction with other mutations. In line with this, (FM 6) harboring p.Q34R mutation in *PARKIN* was also found to be heterozygous for p.T240A novel missense change in the same gene (S1B Fig), however, whether these variants co-segregate with the disease or not, could not be assessed as DNA samples from unaffected family members were not available.

Moreover, two affected siblings (FM 19) (S1A Fig) were heterozygous carriers for two variants in *PARKIN*; p.T240M and p.V380L, a polymorphysim widely reported in various ethnic groups [25, 26]. Even though p.T240M was reported as a disease-causing mutation [19], its pathogenicity remains unconfirmed, since it has been predicted as neutral by two out of four programs (S1 Table) and has been detected in one control. There are conflicting reports with regard to the impact of *PARKIN* (p.V380L) polymorphism on PD risk, however, a recent meta-analytic study demonstrated association of this polymorphism with moderate protection against the disease [26]. Meanwhile, whether this variant exerts the same effect in Saudi population or not, is yet to be known.

Another HGMD-listed mutation reported here, is the p.R98Q mutation of *PARK7*/*DJ1* observed in a heterozygous state in one sporadic case and two affected siblings and their unaffected father (FM 92) displaying an AR from of PD (S1C Fig). Even though it has been classified in HGMD as disease-causing, this mutation is likely to be a polymorphism as suggested by the benign prediction analysis (S1 Table) and its reported presence at a similar frequency in European PD patients and ethnically matching healthy controls [46]. Also this mutation didn’t alter protein stability when expressed in mammalian cells [45, 49]. In contrast, this mutation has been shown to alter PARK7/DJ1 interaction with its binding partners and to reduce its antioxidant activity [50–52]. Therefore, more comprehensive functional analysis is necessary to ascertain the impact of this variant.

The general thinking has been that *PARKIN* or *PINK1*-mediated PD occurs through autosomal recessive inheritance, whereby the presence of homozygous or compound heterozygous mutations is necessary to drive the disease. As most of *PARKIN* and *PINK1* variants, detected in this study, were observed in the patients in heterozygous condition, their clinical significance would be hard to interpret especially when *in vivo* and *in vitro* functional studies are lacking. Intriguingly, although single heterozygous variants are considered insufficient *per se* to cause the disease, subclinical dopamine dysfunction has been shown using functional neuroimaging in asymptomatic individuals heterozygous for *PARKIN*/*PINK1* single mutations [reviewed in [53]]. This observation points towards a possible role of *PARKIN*/*PINK1* heterozygous mutations in the pathogenesis of PD. Beyond monogenic inheritance, the “dual hit” hypothesis, whereby a second hit (be it exposure to environmental toxins, additional mutations in other PD genes or other pathways relevant to the disease), may offer an explanation for some of the heterozygous cases [44, 54].

The current study was initially designed to test only for variants in the coding sequence and intron-exon junctions, thus other genetic events such as exonic rearrangements, copy number alteration and mutations within intron or regulatory regions could be overlooked. To that end we preformed CNV analysis of PD-genes (selected in this study) on a subset of samples. CNV changes were observed in PD patients mainly in *SCNA* and *PARKIN* genes [55]. Our CNV analysis failed to detect any gene dosage alterations in PD related genes apart from *PARKIN*. A homozygous loss that affected exon 7 was detected in affected members in one family (S4D Fig and S3 Table), and a homozygous deletion of exons 3 and 4 was confirmed in a sporadic case (S3 Table), deletions affecting these exons were previously reported [56, 57]. A common loss that affected exon 3, the most frequently reported mutation in *PARKIN* [4], and parts of introns 2 and 3 was observed in 2 families. The two affected siblings from one family were heterozygous for this deletion in addition to the p.T240M point mutation in *PARKIN* (S1A and S4A Figs), in the second family the affected father was homozygous for this deletion while his unaffected son was heterozygous for this deletion (S4B Fig).

We also, evaluated the mRNA expression of PD-common genes (S3 Fig) in 12 samples. Transcripts were present in all samples, except one case (PD-25a) harboring *PARKIN* exon 7 homozygous deletion (S3 Table) in which *PARKIN* transcript was shorter (S3C Fig). These results indicate that mutation(s) in the regulatory elements of the screened genes or in other molecules involved in their transcription process are unlikely to be present at least in the tested samples.

In general, ~82% of reported mutations in PD patients are simple mutations and ~18% are CNV changes [53]. Our comprehensive analysis of PD causative and related genes identified only three point mutation and 2 CNVs. Thus, it seems reasonable to conclude that mutations in the ORF of the screened genes are not a common cause of PD in Saudi population. However, although our findings do not rule out the possibility of the involvement of the screened genes in the development of the disease, as gene expression/dosage may be perturbed, it is tempting to speculate the involvement of still unidentified genes.

## Supporting Information

S1 Fig Pedigrees of PD-familial cases displaying AD/AR mode of inheritance and a sporadic case in which HGMD-listed/novel variants have been identified. (A) The two affected siblings of FM 19 share a reported mutation (p.T240M) and a polymorphism (p.V380L) in *PARKIN*. (B) FM 6 proband and affected father are both compound heterozygous for a novel variant (p.T240A) and a reported mutation (p.Q34R) in *PARKIN*. (C) Two affected siblings and their healthy father share two reported heterozygous variants; (p.R98Q) in *PARK7*/*DJ1* and (p.I723V) in *LRRK2*. (D) Pedigree shows a sporadic occurrence of LOPD in patient (SP-7) harboring two heterozygous variants; p.E195Q in *PARKIN* and p.N551K in *LRRK2*. DNA from unaffected family members is not available. The asterisk denotes novel variant. AAO: age at onset. y: years. # variant with unknown clinical significance.
(TIF)

S2 Fig Multiple sequence alignment of PARKIN and PINK1 homologs from diverse species using ClustalW2. (A and C) Sequence alignment of PINK1 amino acids shows (A) Gly409 to be a highly conserved residue across diverse species, while (C) Gly 189 residue is mapped to a poorly conserved region of human PINK1. RefSeq accession numbers are as follows: Human (NP_115785), Chimpanzee (XP_001164912.2), Macaque (AFI34437), Pig (XP_005665148.1), Mouse (NP_081156.2), Rat (NP_001100164.1), Chicken (XP_423139.3), Zebrafish (NP_001008628.1). (B and D) Sequence alignment of PARKIN amino acids showing conservation of (B) Glu195 among mammals, unlike (D) Thr240 which is of limited conservation. RefSeq accession numbers are as follows: Human (NP_054643), Chimpanzee (XP_001153913), Macaque (ENSMMUT00000028706), Pig (NP_001038068), Mouse (NP_057903), Rat (NP_064478.1), Chicken (XP_419615.3), Zebrafish (NP_001017635).
(TIF)

S3 Fig cDNA amplification of *LRRK2*, *PINK1*, *SNCA*, *PARKIN* and *PARK7*/*DJ1*in a subset of PD samples. Representative electrophoresis images of RT-PCR products of PD-genes co-amplified with *ACTB*. (A) *LRRK2* (top panel) *PINK1* (middle panel) and *SNCA* (bottom panel). (B) *PARK7*/*DJ1*. (C) A short *PARKIN* transcript (~160bp) is detected in PD-25a harboring exon 7 deletion (137bp). PD: patient’s sample. NC: normal control sample.
(TIF)

S4 Fig Pedigrees of the familial cases with aberrant *PARKIN* exon/intron dosage. (A) The two affected siblings of FM 19, share a heterozygous loss of exon 3 and parts of introns 2 and 3. (B) The proband (21-a) is homozygous for multiple deletions (exon 3, intron 4 and parts of introns 2 and 3), whereas his unaffected son (21-b) is heterozygous for exon 3 deletion and partial loss of introns 2 and 3. (C) A heterozygous gain of exon 6 and partial loss of introns 5 and 6 detected in the proband. (D) The affected siblings harbor a homozygous loss of exon 7 and partial loss of introns 6 and 7. AAO: age at onset. y: years. ¥: RNA and DNA samples are available. *: only DNA sample is available. EX: exon. Int: intron. Numbers in bold indicate complete loss and in regular font indicate partial loss. DNA samples from the rest of the family members are not available for evaluation.
(TIF)

S5 Fig *PARKIN* exons 3 and 4 deletions in SP-103. Gel electrophoresis images of the PCR products of either exon 3 (left) or exon 4 (right) co-amplified with *DCC* exon 3 (as an internal control) in SP-103 and a normal control (NC).
(TIF)

S6 Fig *PARKIN* nonsense mutation in PD-110. Part of the sequencing chromatogram of *PARKIN* exon 4 showing homozygous c.532C>T mutation creating a premature stop codon p.Q178X in a sporadic case (PD-110) with EOPD.
(TIF)

S1 File Detailed description of the clinical features of selected families and sporadic cases as well as 3D-structural modelling and cDNA PCR information. (DOCX)

S1 Table Reported sequence variants detected in this study. (DOCX)

S2 Table cDNA PCR primers sequences and product sizes. (DOCX)

S3 Table Summary of the CNV analysis results. (DOCX)
