# Supplementary figures and images for: Parkinson’s Disease in Saudi Patients: A Genetic Study
Source: PLoS One. 2015 Aug 14;10(8):e0135950. doi: 10.1371/journal.pone.0135950 (PMC4537238; doi:10.1371/journal.pone.0135950)

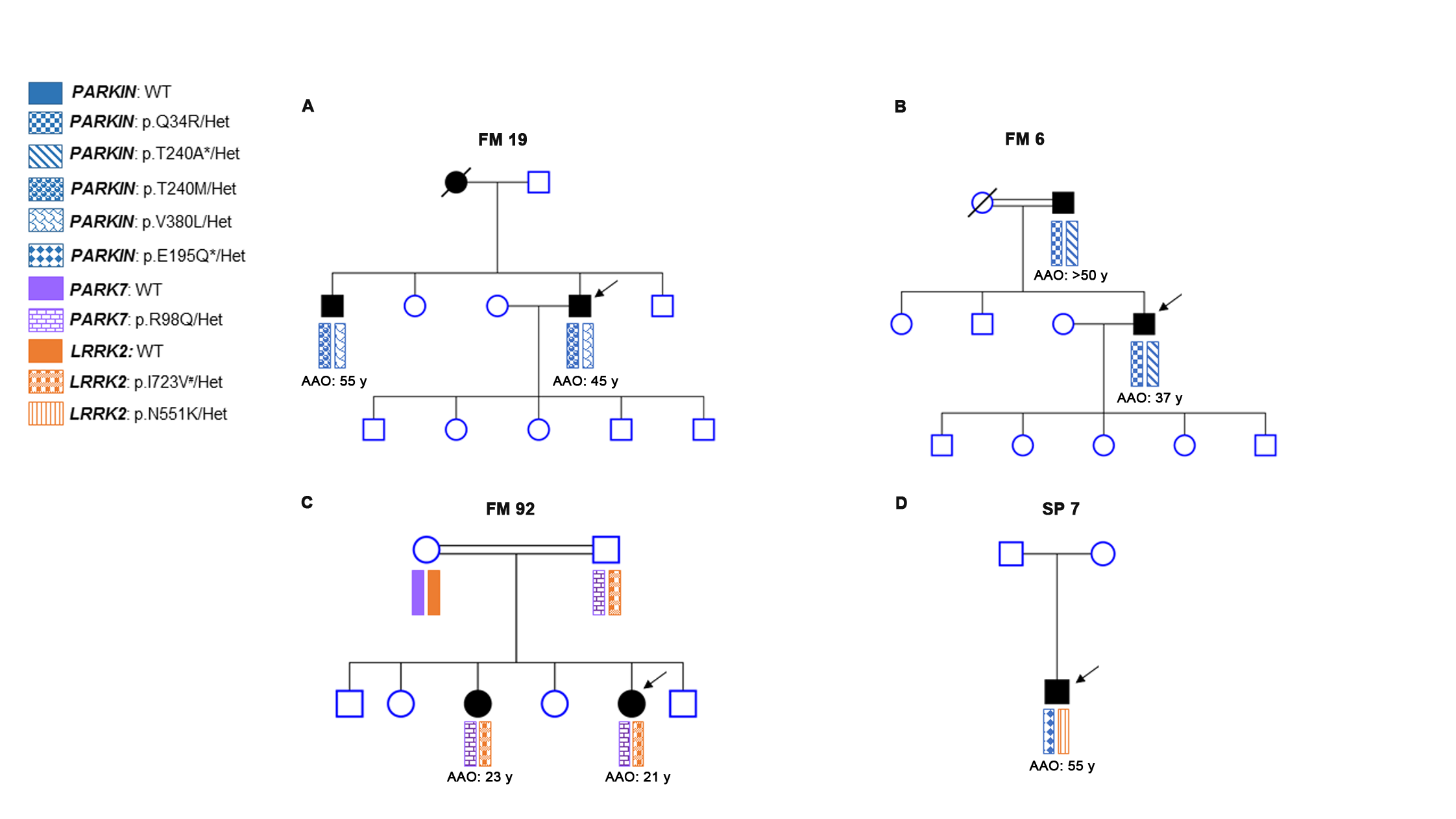

Supplement: S1 Fig — (A) The two affected siblings of FM 19 share a reported mutation (p.T240M) and a polymorphism (p.V380L) in PARKIN. (B) FM 6 proband and affected father are both compound heterozygous for a novel variant (p.T240A) and a reported mutation (p.Q34R) in PARKIN. (C) Two affected siblings and their healthy father share two reported heterozygous variants; (p.R98Q) in PARK7/DJ1 and (p.I723V) in LRRK2. (D) Pedigree shows a sporadic occurrence of LOPD in patient (SP-7) harboring two heterozygous variants; p.E195Q in PARKIN and p.N551K in LRRK2. DNA from unaffected family members is not available. The asterisk denotes novel variant. AAO: age at onset. y: years. # variant with unknown clinical significance. (TIF) [file pone.0135950.s001.tif]

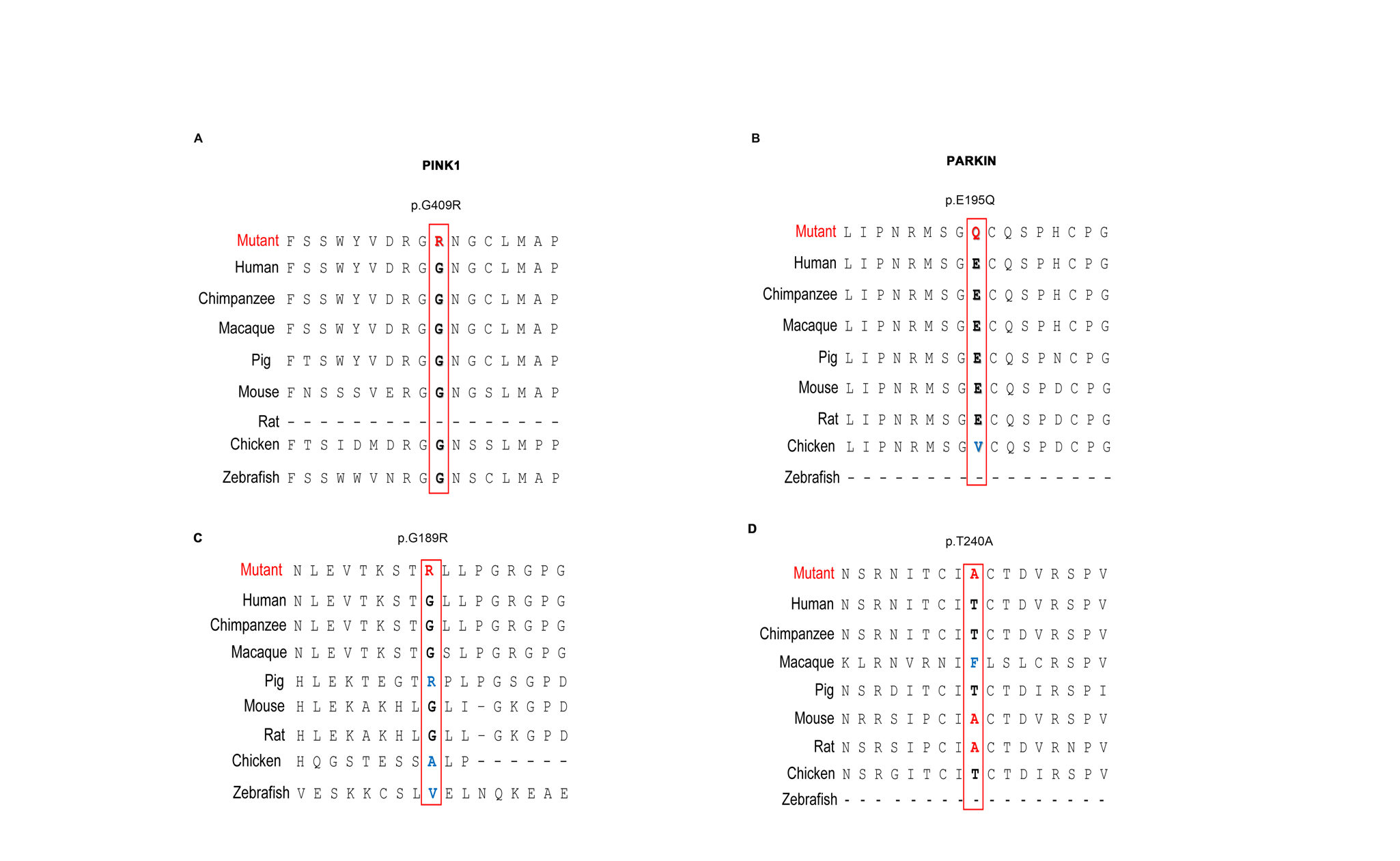

Supplement: S2 Fig — (A and C) Sequence alignment of PINK1 amino acids shows (A) Gly409 to be a highly conserved residue across diverse species, while (C) Gly 189 residue is mapped to a poorly conserved region of human PINK1. RefSeq accession numbers are as follows: Human (NP_115785), Chimpanzee (XP_001164912.2), Macaque (AFI34437), Pig (XP_005665148.1), Mouse (NP_081156.2), Rat (NP_001100164.1), Chicken (XP_423139.3), Zebrafish (NP_001008628.1). (B and D) Sequence alignment of PARKIN amino acids showing conservation of (B) Glu195 among mammals, unlike (D) Thr240 which is of limited conservation. RefSeq accession numbers are as follows: Human (NP_054643), Chimpanzee (XP_001153913), Macaque (ENSMMUT00000028706), Pig (NP_001038068), Mouse (NP_057903), Rat (NP_064478.1), Chicken (XP_419615.3), Zebrafish (NP_001017635). (TIF) [file pone.0135950.s002.tif]

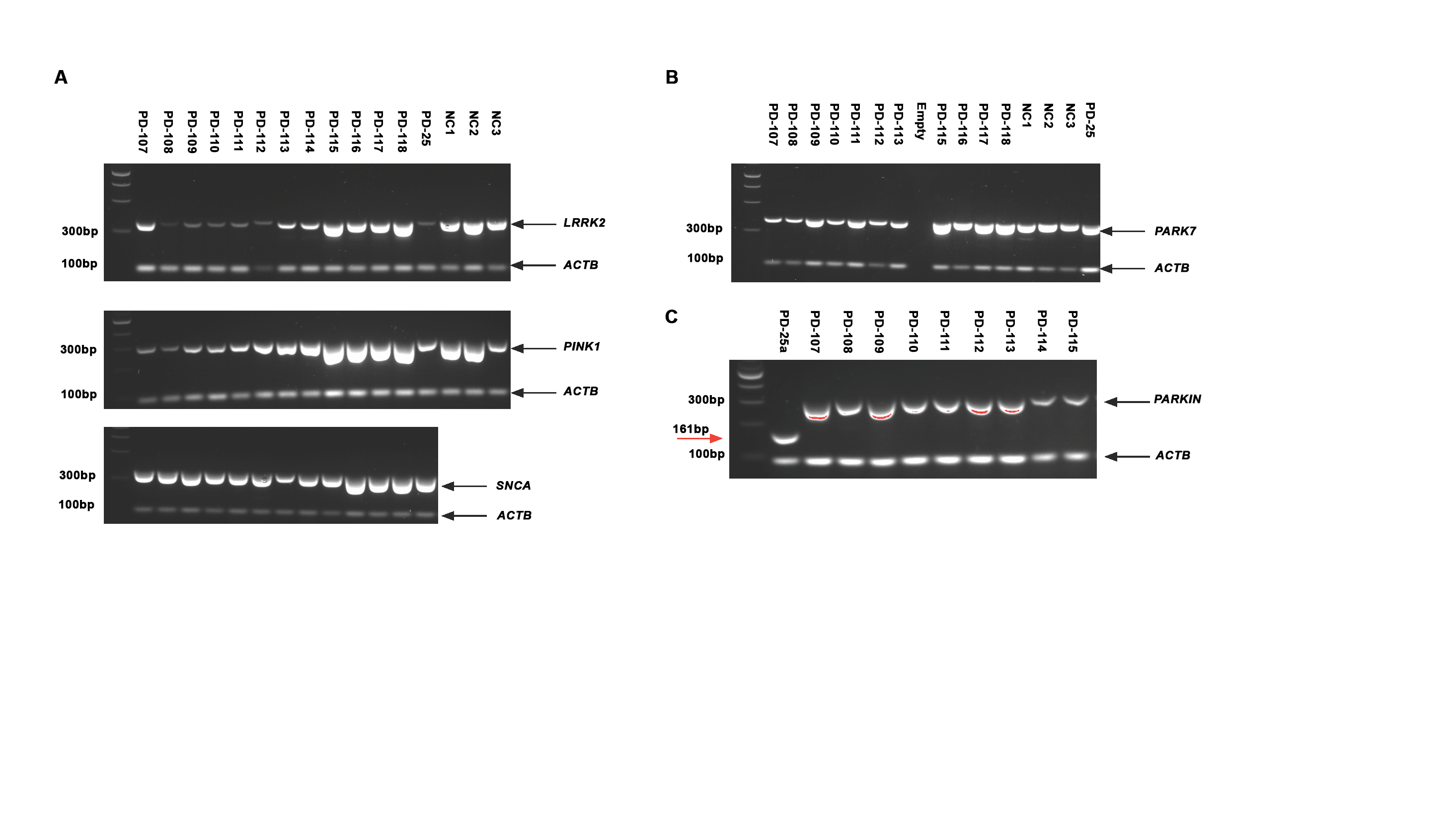

Supplement: S3 Fig — Representative electrophoresis images of RT-PCR products of PD-genes co-amplified with ACTB. (A) LRRK2 (top panel) PINK1 (middle panel) and SNCA (bottom panel). (B) PARK7/DJ1. (C) A short PARKIN transcript (~160bp) is detected in PD-25a harboring exon 7 deletion (137bp). PD: patient’s sample. NC: normal control sample. (TIF) [file pone.0135950.s003.tif]

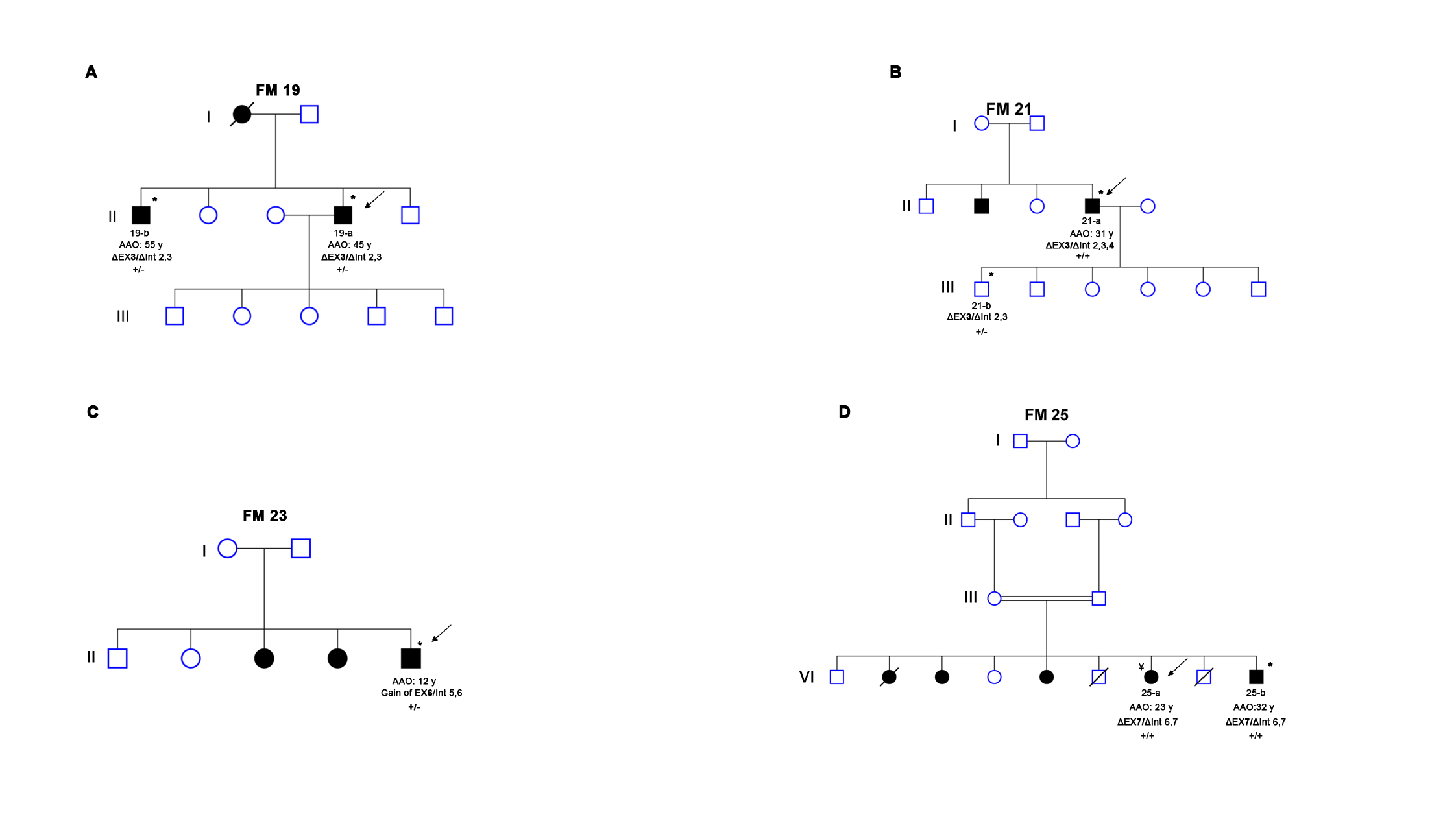

Supplement: S4 Fig — (A) The two affected siblings of FM 19, share a heterozygous loss of exon 3 and parts of introns 2 and 3. (B) The proband (21-a) is homozygous for multiple deletions (exon 3, intron 4 and parts of introns 2 and 3), whereas his unaffected son (21-b) is heterozygous for exon 3 deletion and partial loss of introns 2 and 3. (C) A heterozygous gain of exon 6 and partial loss of introns 5 and 6 detected in the proband. (D) The affected siblings harbor a homozygous loss of exon 7 and partial loss of introns 6 and 7. AAO: age at onset. y: years. ¥: RNA and DNA samples are available. *: only DNA sample is available. EX: exon. Int: intron. Numbers in bold indicate complete loss and in regular font indicate partial loss. DNA samples from the rest of the family members are not available for evaluation. (TIF) [file pone.0135950.s004.tif]

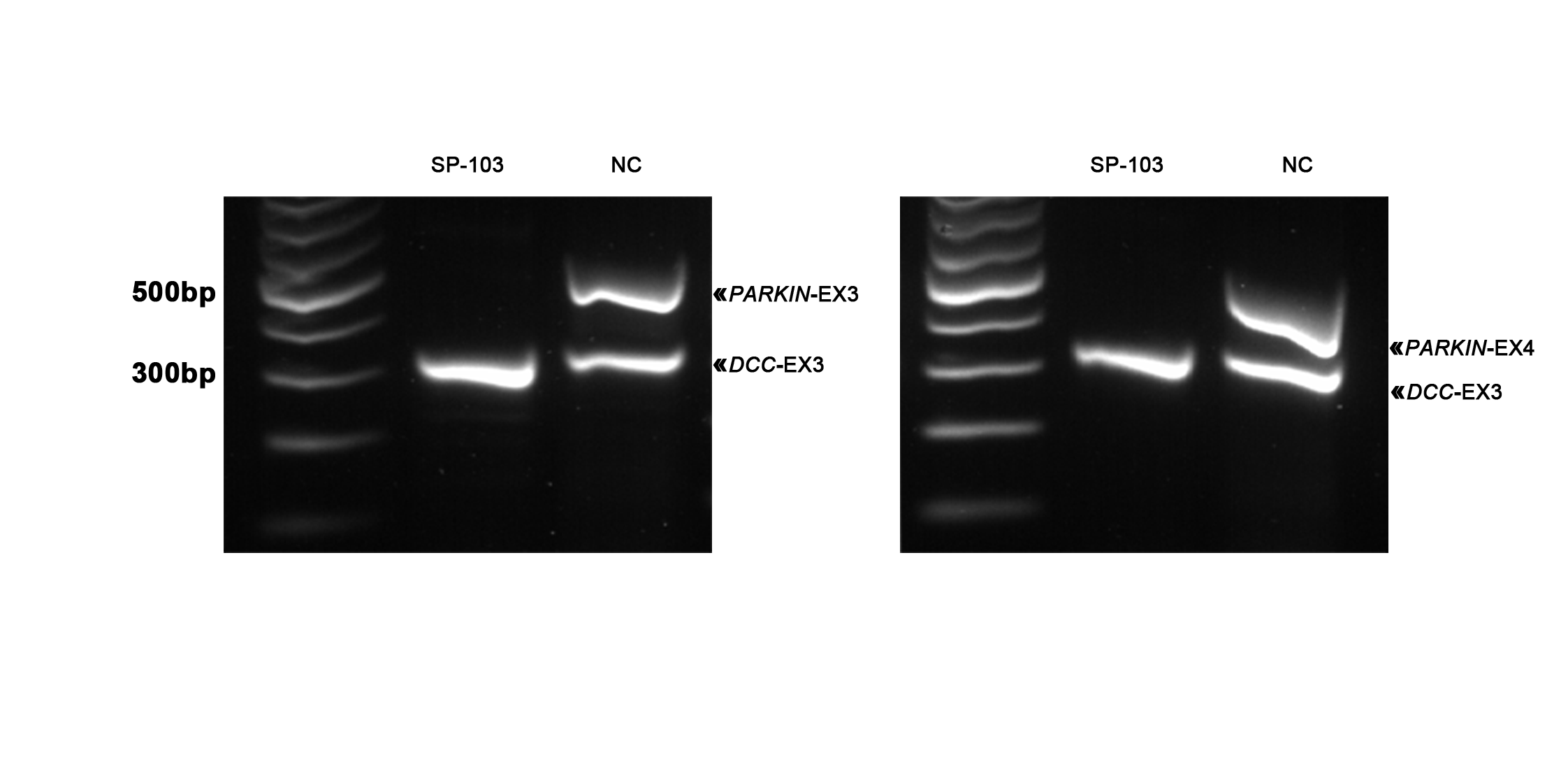

Supplement: S5 Fig — Gel electrophoresis images of the PCR products of either exon 3 (left) or exon 4 (right) co-amplified with DCC exon 3 (as an internal control) in SP-103 and a normal control (NC). (TIF) [file pone.0135950.s005.tif]

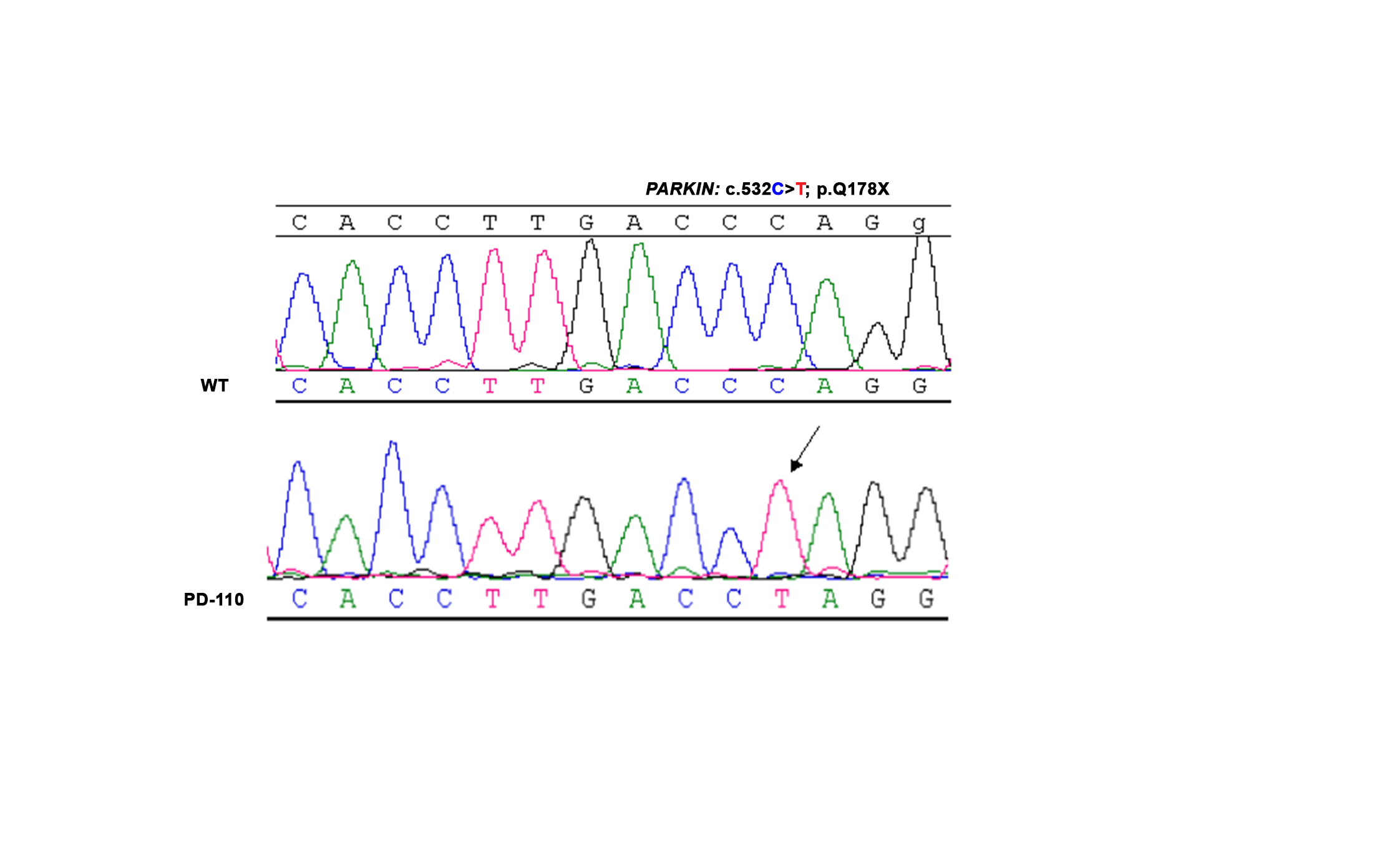

Supplement: S6 Fig — Part of the sequencing chromatogram of PARKIN exon 4 showing homozygous c.532C>T mutation creating a premature stop codon p.Q178X in a sporadic case (PD-110) with EOPD. (TIF) [file pone.0135950.s006.tif]
