# Supplementary material for: Parkinson’s Disease in Saudi Patients: A Genetic Study
Source: PLoS One. 2015 Aug 14;10(8):e0135950. doi: 10.1371/journal.pone.0135950 (PMC4537238; doi:10.1371/journal.pone.0135950)
Supplement: S1 File — (DOCX) [file pone.0135950.s007.docx]

**Supplementary Material**

# **Detailed Clinical features of families and a single sporadic case in which HGMD-listed/novel variants have been detected**

*FM 6*

The proband presented with the disease at 37 years of age. He displayed asymmetric onset, resting tremor, bradykinesia, rigidity, dystonia, and positive levodopa response along with the adverse effects triggered by long-term levodopa therapy, but no gait impairment. As for non-motor symptoms, hallucination was observed but no signs of depression, dementia, pyramidal tract lesions or ataxia were observed. The father had much later onset of the disease (>50 y) with motor symptoms and levodopa response similar to those observed in the proband. Neuropsychiatric symptoms such as depression were observed, but no signs of ataxia or pyramidal tract lesions were reported.

*SP 7*

The individual reported AAO of 55y, presented with asymmetrical onset, resting tremor, bradykinesia, gait impairment, rigidity and beneficial levodopa response along with the adverse effects secondary to long-term use, but no dystonia was reported. In addition, non-motor symptoms and signs of pyramidal tract lesions or ataxia were not observed

*FM 19*

The siblings were born to non-consanguineous parents of whom the deceased mother was reported to be affected. Interestingly, the two siblings have different age of onset; the proband was diagnosed with PD at age 45 years (EOPD) and his sibling at age 55 years (LOPD). Tremor was observed in the sibling with EOPD but was not confirmed in the other one with LOPD. Both patients displayed bradykinesia, rigidity and positive levodopa response along with the adverse effects triggered by long-term levodopa therapy. Gait impairment was only observed in the sibling with LOPD. Other motor symptoms such as dystonia and non-motor symptoms including, hallucination, depression and dementia were absent. Pyramidal signs and ataxia were absent too. The disparate age of disease onset between these siblings could be explained by the presence or absence of other genetic factors, and/or exposure to different environmental factors.

*FM 49*

Both affected members presented with LOPD. The clinical features of the proband (49-a) included cardinal motor symptoms of PD (asymmetrical onset, resting tremor, bradykinesia, rigidity, gait impairment, as well as good response to dopaminergic therapy and the occurrence of the associated treatment-induced dyskinesia, but no report of dystonia) accompanied with hallucinations and dementia. Other motor manifestations including pyramidal signs and ataxia were absent. The affected sibling (49-b) developed similar motor-symptoms (responsive to levodopa treatment) and dystonia, accompanied with hallucinations but no signs of ataxia or pyramidal lesions.

*FM 92*

The siblings were born to consanguineous healthy couple. The proband was diagnosed with EOPD with asymmetrical onset at the age of 21. She displayed the classical motor features of PD (Rigidity, bradykinesia, tremors, dystonia and impaired gait) accompanied with depression, dementia and was responsive to levodopa treatment. Her sibling started exhibiting neurological symptoms at the age of 23. Like the proband, she had asymmetrical limb rigidity with signs of depression and dementia, however, no other motor symptoms were reported and the patient didn’t receive levodopa treatment. No signs of ataxia or pyramidal lesions were observed in both siblings.

*PD 108*

This case was diagnosed with EOPD at age 33 y. He displayed asymmetric onset, resting tremor, bradykinesia and rigidity but no dystonia or gait impairment. Non-motor symptoms such as depression became evident 5 years post the disease onset. Positive levodopa response was sustained for two years before signs of wearing off were apparent.

*PD 110*

This individual was diagnosed with EOPD with asymmetrical onset at the age of 30. She presented with the cardinal motor symptoms (bradykinesia, tremors and rigidity) in addition to dystonia and gait impairment. Neuropsychiatric symptoms including depression, hallucination and dementia were reported early on in the course of the disease in this patient. The patient showed good levodopa response followed by typical signs of wearing off.

**Detailed clinical features for the 2 cases with *PARKIN* exonic deletions**

*FM 25*

The two affected siblings enrolled in this study (25-a and 25-b) were born to a consanguineous couple and have another 3 affected siblings of which is deceased and the other two were not available for recruitment . The proband and her sibling were both diagnosed with EOPD at 23 and 32 years of age, respectively. The proband (25-a) had asymmetric onset of symptoms, tremors, bradykinesia, rigidity and dystonia with normal gait. She also has positive levodopa response along with the adverse effects triggered by long-term use. No signs of neuropsychiatric symptoms or pyramidal lesions were observed. Unlike the proband, the affected sibling (25-b) did not display asymmetric onset, he presented with the classical motor features such as; tremors, bradykinesia, rigidity and impaired gait but no dystonia. Also depression was reported but no reports on levodopa treatment outcome.

*SP 103*

This sporadic case presented with EOPD at age 20 y. He presented with non-dominant side onset with bradykinesia, rigidity, impaired gait and positive levodopa response but no tremors or dystonia. Non-motor symptoms including depression and hallucination were reported.

# **3D-structural modelling of PINK1, PARKIN and their mutant versions**

5 protein models were predicted for each the wild-type PINK1 (PINK1^wt^) and the mutant PINK1 (PINK1^mut^), however, only the ones with the best C-and Z-scores were considered. The C- and the Z-scores are -2.43, -5.35 and -2.03, -5.08 for PINK1^wt^ and PINK1^mut^ , respectively. The refined models were further validated using ERRAT score, Ramachandran plot and RMS. ERRAT scores were 90.9 and 82 for PINK1^wt^ and PINK1^mut^ , respectively. Ramachandran distribution plot for PINK1^wt^  locates 78.10% of the residues in the favored region, 14.3% in the allowed region and 7.6% in the outlier region, while the distribution for PINK1^mut^  places 75.30% of the residues in the favored region, 15.7% in the allowed region and 9.0% in the outlier region. The estimated RMS value is -8.274 Å indicating a high degree of dissimilarity between the wild-type and mutant PINK1. Pathogenicity and loss of protein stability were predicted for this mutation by PMUT and I-Mutant softwares [[1](#_ENREF_1), [2](#_ENREF_2)].

PARKIN^WT^ and PARKIN^mut^ were modeled on PARKIN template structure from (RCSB/PDB-ID# 4K95) spanning amino acids (141-465). The prediction analysis generated 10 optimal models for each PARKIN^WT^ and PARKIN^mut^. Best models were selected on the basis of MOLPDF and DOPE scores. PARKIN^WT^ MOLPDF and DOPE scores were 7370.7891 and 38557.02344, respectively, while the corresponding scores for PARKIN^mut^ were 3452.457 and -37111.02344. Both selected models were further evaluated on the basis of energy. Local energy plot and ERRAT graph was also constructed. Ramachandran plot for PARKIN^WT^ gave 94.4% residues in the favorably accepted region, 4.5% in the allowed region and 1.1% in the outlier region. As for PARKIN^mut^, 93.1% of the residues were placed in the favored region, 5.2% in the allowed region and 1.7% in the outlier region.. The estimated RMS value is 0.776 Å indicating a very low degree of dissimilarity between the PARKIN^WT^ and mutant PARKIN^mut^. PMUT predicts p.E195Q as being neutral, whereas I-Mutant predicts this substitution to decrease protein stability.

**cDNA amplification primers and PCR conditions**

For each sample second strand cDNA was amplified using 1ul of template cDNA, 2.5µl 10X PCR buffer, 2µl dNTPs (at a final concentration of 0.2mM) 0.7µl of forward and reverse primers (at a final concentration of 0.28mM) and 0.3µl of forward and reverse reference gene primers (at a final concentration of 0.12mM), 0.2µl of Taq polymerase enzyme and the reaction volume was made up to 25µl using nuclease-free water. The thermal cycling parameters were as follows: 15 minutes of initial denaturation at 95°C; 39 cycles of 60 seconds at 95°C, 60 seconds of annealing at 50/52/58°C with 60 seconds of extension at 72°C.

**S2 Table. cDNA PCR primers sequences and product sizes.**

| **Description** | **Gene/Exons covered** | **Transcript-ID** | **Sequence 5'-3'** | **Product size (bp)** |
| --- | --- | --- | --- | --- |
| RtLRRK2X23-24F | *LRRK2*/Ex23-25 | ENST00000298910 | AGCTATGTGAAACTCTGAAG | 311 |
| RtLRRK2X25R |  |  | CTTCAGTTCCTTCAGTCTC |  |
| RtDJ1X4F | *PARK7*/*DJ1*/Ex4-7 | ENST00000338639 | CATATGATGTGGTGGTTCTAC | 335 |
| RtDJ1X7R |  |  | CACCTCCTTGCCATTCAG |  |
| RtPINK1X2F | *PINK1*/Ex2-4 | ENST00000321556 | ACCAGGAGAAGGGCAGGAG | 297 |
| RtPINK1X4R |  |  | AGCACATCAGGGTAGTCGAC |  |
| RtPARK2X6F | *PARKIN*/Ex6-9 | ENST00000366898 | TCGCAACAAATAGTCGGAACA | 298 |
| RtPARK2X9R |  |  | CCCATCTGCAGGACACACT |  |
| RtSNCAX2-3F | *SNCA*/Ex2-6 | ENST00000394986 | GTTCTCTATGTAGGCTCC | 307 |
| RtSNCAX6R |  |  | CAGGTTCGTAGTCTTGAT |  |
| RtB-ACTIN F | *ACTB*/Ex5-6 | ENST00000331789 | ATGCAGAAGGAGATCACTGC | 87 |
| RtB-ACTIN R |  |  | GATCCACACGGAGTACTTGC |  |

**S3 Table. Summary of the CNV analysis results.**

| **GENES/NCBI-ID** | **Sample ID** | **Disease status** | **FM/SP** | **TYPE** | **CHROMSOME** | **FROM** | **TO** | **EXON / INTRON AFFECTED** | **CN STATE** | **Validation** |
| --- | --- | --- | --- | --- | --- | --- | --- | --- | --- | --- |
| *PARKIN/* NM_004562 | 19-a | affected | FM | LOSS | 6 | 162643775 | 162813995 | Exon 3/ intron 2* and 3* | 1 | No RNA |
|  | 19-b | affected sibling of 19-a | FM | LOSS | 6 | 162643775 | 162813995 | Exon 3/ intron 2* and 3* | 1 | No RNA |
|  | 21-a | affected | FM | LOSS | 6 | 162582788 | 162587775 | Intron 4 | 0 | NA |
|  | 21-a | affected | FM | LOSS | 6 | 162638345 | 162754162 | Exon 3/ intron 2* and 3* | 0 | No RNA |
|  | 21-b | unaffected son of 21-a | FM | LOSS | 6 | 162638345 | 162754162 | Exon 3/ intron 2* and 3* | 1 | No RNA |
|  | 25-a | affected | FM | LOSS | 6 | 162187069 | 162281019 | Exon 7 / intron 6* and 7* | 0 | Confirmed/Shorter transcript |
|  | 25-b | affected sibling of 25-a | FM | LOSS | 6 | 162187069 | 162280309 | Exon 7 / intron 6* and 7* | 0 | No RNA |
|  | FM-23 | no sample from family members | FM | GAIN | 6 | 162296841 | 162424430 | Exon 6/ intron 5* and 6* | 4 | No RNA |
|  | SP-103 | affected | SP | LOSS | 6 | 162618047 | 162810362 | Exon 3 and 4 / intron 2*,3 and 4* | 0 | Confirmed/PCR |

The changes listed here are not present in database of genomic variants (DGV), but regions overlap with reported CNVs. CN state; Copy number state was scored as 0=homozygous loss, 1 heterozygous loss, 3 and above gain of copies, 2 is normal copy. * Denotes partial loss, FM; familial, SP; sporadic.

**References**

1. Capriotti E, Fariselli P, Casadio R. I-Mutant2.0: predicting stability changes upon mutation from the protein sequence or structure. Nucleic acids research. 2005 33(Web Server issue):W306-10.

2. Ferrer-Costa C, Gelpi JL, Zamakola L, Parraga I, de la Cruz X, Orozco M. PMUT: a web-based tool for the annotation of pathological mutations on proteins. Bioinformatics (Oxford, England). 2005 21(14):3176-8.

**Web references**

ClustalW2, <http://www.ebi.ac.uk/Tools/msa/clustalw2/>
