# Supplementary material for: Parkinson’s Disease in Saudi Patients: A Genetic Study
Source: PLoS One. 2015 Aug 14;10(8):e0135950. doi: 10.1371/journal.pone.0135950 (PMC4537238; doi:10.1371/journal.pone.0135950)
Supplement: S1 Table — (DOCX) [file pone.0135950.s008.docx]

**S1 Table. Reported sequence variants detected in this study**

|  |  |  | | |  |  |  | |  | |  | |  | |  |  |  |
| --- | --- | --- | --- | --- | --- | --- | --- | --- | --- | --- | --- | --- | --- | --- | --- | --- | --- |
|  | **Variant** | | | | |  | **Prediction tools** | | | | | | |  | |  |  |
| **Gene/PCR primer/position** | **cDNA** | | **Protein** | **Ref.No (dbSNP/HGMD)** | | **HGMD class** | **MutationTaster** | **PolyPhen-2** | | **SIFT** | | **PROVEAN** | | **Het/Homo** | | **FM/SP** | ***Frequency** |
| *PARKIN*/Exon2 | c.101A>G | | Q34R | rs148851677/CM0066954 | | DM | disease causing | probably damaging | | damaging | | Neutral | | 1/0 | | 0/1 | 1/98 |
| *PARKIN*/Exon6 | c.719C>T | | T240M | rs137853054/CM030926 | | DM | polymorphism | possibly damaging | | damaging | | Neutral | | 2/0 | | 2/0 | 1/96 |
| *PINK1*/Exon4 | c.938C>T | | T313M | rs74315359/CM056681 | | DM | disease causing | possibly damaging | | damaging | | Deleterious | | 0/1 | | 0/1 | n.a. |
| *PINK1*/Exon7 | c.1426G>A | | E476K | rs115477764 /CM042455 | | DM | polymorphism | benign | | tolerated | | Neutral | | 1/0 | | 0/1 | 0/96 |
| *PARK7*/Exon5 | c.293G>A | | R98Q | rs71653619/ CM032051 | | DM | polymorphism | benign | | tolerated | | Neutral | | 2/0 | | 1/1 | n.a. |
| *LRRK2*/Exon14 | c.1653C>G T | | N551K | rs7308720/CM104618 | | DP | _ | _ | | _ | | _ | | 3/0 | | 1/2 | n.a. |
| *UCHL1*/Exon3 | c.53C>A | | S18Y | rs5030732/CM994452 | | DP | _ | _ | | _ | | _ | | 21/1 | | 5/17 | n.a. |
| *PARKIN*/Exon4 | c.500G>A | | S167N | rs1801474/CM992652 | | DP | _ | _ | | _ | | _ | | 4/0 | | 2/2 | n.a. |
| *PARKIN*/Exon10 | c.1138G>C | | V380L | rs1801582 /CM032983 | | DP | _ | _ | | _ | | _ | | 24/0 | | 12/10 | 20/96 |
| *SNCA*/Exon3/intron3 | c.163+162T>A | | _ | rs1442149 | | _ | _ | _ | | _ | | _ | | 5/0 | | 3/2 | n.a. |
| *SNCA*/Exon4/intron4 | c.306+66G>A | | _ | rs10005233 | | _ | _ | _ | | _ | | _ | | 18/12 | | 11/19 | n.a. |
| *SNCA*/Exon 6/intron5 | c.391-54A>G | | _ | rs6842093 | | _ | _ | _ | | _ | | _ | | 17/0 | | 7/10 | n.a. |
| *PARKIN*/Exon2/intron2 | c.171+25T>C | | _ | rs2075923 | | _ | _ | _ | | _ | | _ | | 8/11 | | 9/10 | n.a. |
| *PARKIN*/Exon2 | c.111G>A | | P37P | rs77795533 | | _ | _ | _ | | _ | | _ | | 3/0 | | 0/3 | n.a. |
| *PARKIN*/Exon3/intron2 | c.413-20T>C | | _ | rs4709583 | | _ | _ | _ | | _ | | _ | | 2/15 | | 5/12 | n.a. |
| *PARKIN*/Exon5/intron5 | c.618+72T>C | | _ | rs112448533 | | _ | _ | _ | | _ | | _ | | 1/0 | | 0/1 | n.a. |
| *PARKIN*/Exon8/intron7 | c.872-68C>G | | _ | rs3765475 | | _ | _ | _ | | _ | | _ | | 4/35 | | 16/23 | n.a. |
| *PARKIN*/Exon8/intron8 | c.933+48C>T | | _ | rs10945756 | | _ | _ | _ | | _ | | _ | | 22/7 | | 12/17 | n.a. |
| *PARKIN*/Exon8/intron7 | c.872-35G>A | | _ | rs3765474 | | _ | _ | _ | | _ | | _ | | 7/34 | | 16/25 | n.a. |
| *UCHL1*/Exon1-2/5'UTR | c.-16C>T | | _ | rs9321 | | _ | _ | _ | | _ | | _ | | 3/1 | | 1/3 | n.a. |
| *UCHL1*/Exon1-2/5'UTR | c.-24A>G | | _ | rs11556271 | | _ | _ | _ | | _ | | _ | | 3/1 | | 1/3 | n.a. |
| *UCHL1*/Exon1-2/intron2 | c.45+6T>C | | _ | rs11556273 | | _ | _ | _ | | _ | | _ | | 4/0 | | 0/4 | n.a. |
| *UCHL1*/Exon1-2/intron2 | c.45+19C>A | | _ | rs11556272 | | _ | _ | _ | | _ | | _ | | 4/0 | | 0/4 | n.a. |
| *UCHL1*/Exon8/ intron8 | c.585+117C>T | | _ | rs3775256 | | _ | _ | _ | | _ | | _ | | 17/1 | | 2/16 | n.a. |
| *UCHL1*/Exon8/intron7 | c.527-74C>A | | _ | rs71608068 | | _ | _ | _ | | _ | | _ | | 2/0 | | 0/2 | n.a. |
| *UCHL1*/Exon8 | c.558C>T | | G186G | rs116680633 | | _ | _ | _ | | _ | | _ | | 1/1 | | 2/0 | n.a. |
| *UCHL1*/Exon8/intron7 | c.527-131C>G | | _ | rs3756001 | | _ | _ | _ | | _ | | _ | | 5/0 | | 0/5 | n.a. |
| *UCHL1*/Exon9/intron8 | c.586-35G>A | | _ | rs116293750 | | _ | _ | _ | | _ | | _ | | 1/1 | | 2/0 | n.a. |
| *PINK1*/Exon2/intron1 | c.388-7A>G | | _ | rs2298298 | | _ | _ | _ | | _ | | _ | | 0/27 | | 5/22 | n.a. |
| *PINK1*/Exon5/intron4 | c.960-5G>A | | _ | rs3131713 | | _ | _ | _ | | _ | | _ | | 0/18 | | 6/12 | n.a. |
| *PINK1*/Exon8 | c.1562A>C | | N521T | rs1043424 | | _ | _ | _ | | _ | | _ | | 13/0 | | 4/9 | n.a. |
| *PINK1*/Exon8/3'UTR | c.*37A>T | | _ | rs686658 | | _ | _ | _ | | _ | | _ | | 0/58 | | 18/40 | n.a. |
| *LRRK2*/Exon1 | c.149G>A | | R50H | rs2256408 | | _ | _ | _ | | _ | | _ | | 0/71 | | 26/45 | n.a. |
| *LRRK2*/Exon2/intron1 | c.152-56G>A | | _ | rs2723273 | | _ | _ | _ | | _ | | _ | | 0/7 | | 2/5 | n.a. |
| *LRRK2*/Exon2/intron2 | c.237+100A>G | | _ | rs2256286 | | _ | _ | _ | | _ | | _ | | 1/51 | | 19/32 | n.a. |
| *LRRK2*/Exon3/intron3 | c.347+45T>C | | _ | rs1352879 | | _ | _ | _ | | _ | | _ | | 0/48 | | 16/32 | n.a. |
| *LRRK2*/Exon4/intron4 | c.456+38A>T | | _ | rs2131088 | | _ | _ | _ | | _ | | _ | | 0/3 | | 1/2 | n.a. |
| *LRRK2*/Exon5 | c.457T>C | | L152L | rs10878245 | | _ | _ | _ | | _ | | _ | | 16/11 | | 6/21 | n.a. |
| *LRRK2*/Exon6/intron5 | c. 572-127T>C | | _ | rs6581622 | | _ | _ | _ | | _ | | _ | | 11/4 | | 8/7 | n.a. |
| *LRRK2*/Exon6/intron5 | c.572-82A>G | | _ | rs11564187 | | _ | _ | _ | | _ | | _ | | 11/2 | | 6/7 | n.a. |
| *LRRK2*/Exon8/intron7 | c.839-160C>T | | _ | rs732374 | | _ | _ | _ | | _ | | _ | | 11/7 | | 7/11 | n.a. |
| *LRRK2*/Exon9/intron8 | c.1102-10C>A | | _ | rs7955902 | | _ | _ | _ | | _ | | _ | | 14/3 | | 4/13 | n.a. |
| *LRRK2*/Exon11/intron11 | c.1288+130G>A | | _ | rs7969677 | | _ | _ | _ | | _ | | _ | | 8/0 | | 3/5 | n.a. |
| *LRRK2*/Exon12/intron11 | c.1289-175A>T | | _ | rs11564209 | | _ | _ | _ | | _ | | _ | | 0/9 | | 3/6 | n.a. |
| *LRRK2*/Exon14/intron13 | c.1544-54A>G | | _ | rs10784461 | | _ | _ | _ | | _ | | _ | | 25/8 | | 7/26 | n.a. |
| LRRK2/Exon15/intron14 | c.1657-92A>T | | _ | rs191107526 | | _ | _ | _ | | _ | | _ | | 4/0 | | 1/3 | n.a. |
| *LRRK2*/Exon18 | c.2167A>G | | I723V | rs10878307 | | _ | _ | _ | | _ | | _ | | 21/3 | | 10/14 | n.a. |
| *LRRK2*/Exon19/intron18 | c.2242-22C>T | | _ | rs36220738 | | _ | _ | _ | | _ | | _ | | 0/1 | | 1/0 | n.a. |
| *LRRK2*/Exon20/intron20 | c.2689+145A>G | | _ | rs11564270 | | _ | _ | _ | | _ | | _ | | 2/0 | | 2/0 | n.a. |
| *LRRK2*/Exon20 | c.2594C>T | | S865F | rs142700458 | | _ | _ | _ | | _ | | _ | | 20/0 | | 7/13 | n.a. |
| LRRK2/Exon22/intron21 | c.2809-150C>T | | _ | rs4768226 | | _ | _ | _ | | _ | | _ | | 1/0 | | 0/1 | n.a. |
| LRRK2/Exon22 | c.2857T>C | | L953L | rs7966550 | | _ | _ | _ | | _ | | _ | | 0/1 | | 1/0 | n.a. |
| *LRRK2*/Exon23/intron23 | c.3096+142C>T | | _ | rs11175922 | | _ | _ | _ | | _ | | _ | | 25/2 | | 7/20 | n.a. |
| LRRK2/Exon28 | c.3808C>T | | L1270L | rs201775131 | | _ | _ | _ | | _ | | _ | | 9/0 | | 2/7 | n.a. |
| *LRRK2*/Exon30/intron29 | c.4190-62A>T | | _ | rs7305344 | | _ | _ | _ | | _ | | _ | | 10/4 | | 2/12 | n.a. |
| *LRRK2*/Exon33/intron33 | c.4827+83A>G | | _ | rs721713 | | _ | _ | _ | | _ | | _ | | 0/10 | | 2/8 | n.a. |
| *LRRK2*/Exon34 | c.4872C>A | | G1624G | rs1427263 | | _ | _ | _ | | _ | | _ | | 34/7 | | 15/26 | n.a. |
| *LRRK2*/Exon34 | c.4911A>G | | K1637K | rs11176013 | | _ | _ | _ | | _ | | _ | | 40/9 | | 12/37 | n.a. |
| LRRK2/Exon35/intron34 | c.5016-51A>T | | _ | rs10878368 | | _ | _ | _ | | _ | | _ | | 12/13 | | 8/17 | n.a. |
| *LRRK2*/Exon35/intron35 | c.5170+93G>A | | _ | rs17444028 | | _ | _ | _ | | _ | | _ | | 7/5 | | 5/7 | n.a. |
| *LRRK2*/Exon35/intron35 | c.5170+23T>A | | _ | rs7307276 | | _ | _ | _ | | _ | | _ | | 18/14 | | 13/19 | n.a. |
| *LRRK2*/Exon40/intron40 | c.5948+48C>T | | _ | rs2404834 | | _ | _ | _ | | _ | | _ | | 14/0 | | 7/7 | n.a. |
| *LRRK2*/Exon42/intron42 | c.6280+109T>G | | _ | rs17461992 | | _ | _ | _ | | _ | | _ | | 8/2 | | 3/7 | n.a. |
| *LRRK2*/Exon43 | c.6324G>A | | E2108E | rs10878405 | | _ | _ | _ | | _ | | _ | | 10/0 | | 4/6 | n.a. |
| *LRRK2*/Exon45 | c.6764A>T | | K2255M | rs200762374 | | _ | _ | _ | | _ | | _ | | 1/0 | | 0/1 | n.a. |
| *LRRK2/*Exon48/inron47 | c.7029-89_7029-88insCATG | | _ | rs35456667 | | _ | _ | _ | | _ | | _ | | 0/3 | | 2/1 | n.a. |
| *LRRK2/*Exon48/inron47 | c.7029-8C>T | | _ | rs202087438 | | _ | _ | _ | | _ | | _ | | 2/0 | | 2/0 | n.a. |
| *LRRK2/*Exon48 | c.7155A>G | | G2385G | rs33962975 | | _ | _ | _ | | _ | | _ | | 3/1 | | 4/0 | n.a. |
| *LRRK2*/Exon49 | c.7190T>C | | M239T | rs3761863 | | _ | _ | _ | | _ | | _ | | 31/14 | | 15/30 | n.a. |
| *LRRK2*/Exon50/intron49 | c.7391-44T>C | | _ | rs3789329 | | _ | _ | _ | | _ | | _ | | 11/0 | | 3/8 | n.a. |
| *LRRK2*/Exon50/intron50 | c.7462+128A>G het | | _ | rs3789328 | | _ | _ | _ | | _ | | _ | | 2/0 | | 0/2 | n.a. |
| *LRRK2*/Exon51/3'UTR | c.*4353T>C | | _ | rs66737902 | | _ | _ | _ | | _ | | _ | | 13/0 | | 7/6 | n.a. |
| *GIGYF2*/Exon5/5'UTR | c.-4A>C | | _ | rs11555646 | | _ | _ | _ | | _ | | _ | | 38/13 | | 7/44 | n.a. |
| *GIGYF2*/Exon12/intron11 | c.713-172A>G | | _ | rs13393621 | | _ | _ | _ | | _ | | _ | | 1/0 | | 0/1 | n.a. |
| *GIGYF2*/Exon16/intron15 | c.1480-68T>G | | _ | rs2305139 | | _ | _ | _ | | _ | | _ | | 21/0 | | 2/19 | n.a. |
| *GIGYF2*/Exon17 | c.1716G>T | | A572A | rs114498122 | | _ | _ | _ | | _ | | _ | | 6/0 | | 1/5 | n.a. |
| *GIGYF2*/Exon22/intron21 | c.2209-69T>G | | _ | rs1078323 | | _ | _ | _ | | _ | | _ | | 7/0 | | 0/7 | n.a. |
| *GIGYF2*/Exon23/intron22 | c.2371-135A>G | | _ | rs2305141 | | _ | _ | _ | | _ | | _ | | 3/0 | | 0/3 | n.a. |
| *GIGYF2*/Exon26 | c.2940A>G | | Q980Q | rs3816334 | | _ | _ | _ | | _ | | _ | | 27/15 | | 3/34 | n.a. |
| *GIGYF2*/Exon29/intron28 | c.3461-9G>A | | _ | rs2305137 | | _ | _ | _ | | _ | | _ | | 25/11 | | 6/30 | n.a. |
| *GIGYF2*/Exon29 | c.3626T>A | | L1209Q | rs114013774 | | _ | _ | _ | | _ | | _ | | 1/0 | | 0/1 | n.a. |
| *GIGYF2*/Exon29 | c.3651G>A | | P1217P | rs12328151 | | _ | _ | _ | | _ | | _ | | 2/0 | | 0/2 | n.a. |
| *GIGYF2*/Exon31/intron30 | c.3833-48G>A | | _ | rs1947105 | | _ | _ | _ | | _ | | _ | | 22/5 | | 2/25 | n.a. |
| *FBXO7*/Exon1/intron1 | c.122+116C>T | | _ | rs8136485 | | _ | _ | _ | | _ | | _ | | 1/1 | | 0/2 | n.a. |
| *FBXO7*/Exon1/intron1 | c.122+119G>T | | _ | rs11703157 | | _ | _ | _ | | _ | | _ | | 3/2 | | 0/5 | n.a. |
| *FBXO7*/Exon2 | c.345G>A | | M115 I | rs11107 | | _ | _ | _ | | _ | | _ | | 21/4 | | 6/19 | n.a. |
| *FBXO7*/Exon6/intron5 | c.872-75T>C | | _ | rs738982 | | _ | _ | _ | | _ | | _ | | 15/7 | | 5/17 | n.a. |
| *FBXO7*/Exon6 | c.949C>T | | L317L | rs9726 | | _ | _ | _ | | _ | | _ | | 23/6 | | 5/24 | n.a. |
| *VPS35*/Exon2/intron2 | c.102+108C>T | | _ | rs695997 | | _ | _ | _ | | _ | | _ | | 0/13 | | 1/12 | n.a. |
| *VPS35*/Exon4/intron4 | c.323+98C>T | | _ | rs700581 | | _ | _ | _ | | _ | | _ | | 0/14 | | 2/12 | n.a. |
| *VPS35*/Exon5/intron5 | c.506+54T>C | | _ | rs700582 | | _ | _ | _ | | _ | | _ | | 18/28 | | 7/39 | n.a. |
| *VPS35*/Exon10 | c.1042G>T | | V348L | rs140265194 | | _ | _ | _ | | _ | | _ | | 54/0 | | 8/46 | n.a. |
| *VPS35*/Exon12/intron12 | c.1524+41G>C | | _ | rs4966616 | | _ | _ | _ | | _ | | _ | | 0/2 | | 1/1 | n.a. |
| *VPS35*/Exon12/intron12 | c.1524+115A>G | | _ | rs36310 | | _ | _ | _ | | _ | | _ | | 0/6 | | 0/6 | n.a. |
| *VPS35*/Exon14/intron13 | c.1648-24T>C | | _ | rs2304492 | | _ | _ | _ | | _ | | _ | | 36/22 | | 9/50 | n.a. |
| *VPS35*/Exon15 | c.1938C>T | | H646H | rs168745 | | _ | _ | _ | | _ | | _ | | 8/62 | | 8/62 | n.a. |
| *PARK7*/*DJ1*/Exon2/intron1 | c.-22C>T | | _ | rs11548933 | | _ | _ | _ | | _ | | _ | | 2/0 | | 0/2 | n.a. |
| *PARK7*/*DJ1*/Exon5/intron4 | c.253-98G>A | | _ | rs6703670 | | _ | _ | _ | | _ | | _ | | 7/1 | | 2/6 | n.a. |
| *PARK7*/*DJ1*/Exon5/intron4 | c.253-31C>T | | _ | rs7534132 | | _ | _ | _ | | _ | | _ | | 6/1 | | 2/5 | n.a. |
| *PARK7*/*DJ1*/Exon6/intron5 | c.323-14A>G | | _ | rs72854882 | | _ | _ | _ | | _ | | _ | | 5/2 | | 1/6 | n.a. |

Key: HGMD, The Human gene mutation database; DM, disease-causing mutation; DP, disease-associated polymorphism; Heterozygous, Het; Homozygous, Hom; Familial, FM; Sporadic, SP; Not available, n.a. * Frequency: (n) of control carriers/ total (n) of controls.
