# Supplementary material for: Parkinson’s Disease in Saudi Patients: A Genetic Study
Source: PLoS One. 2015 Aug 14;10(8):e0135950. doi: 10.1371/journal.pone.0135950 (PMC4537238; doi:10.1371/journal.pone.0135950)
Supplement: S2 Table — (DOCX) [file pone.0135950.s009.docx]

| **Description** | **Gene/Exons covered** | **Transcript-ID** | **Sequence 5'-3'** | **Product size (bp)** |
| --- | --- | --- | --- | --- |
| RtLRRK2X23-24F | *LRRK2*/Ex23-25 | ENST00000298910 | AGCTATGTGAAACTCTGAAG | 311 |
| RtLRRK2X25R |  |  | CTTCAGTTCCTTCAGTCTC |  |
| RtDJ1X4F | *PARK7*/*DJ1*/Ex4-7 | ENST00000338639 | CATATGATGTGGTGGTTCTAC | 335 |
| RtDJ1X7R |  |  | CACCTCCTTGCCATTCAG |  |
| RtPINK1X2F | *PINK1*/Ex2-4 | ENST00000321556 | ACCAGGAGAAGGGCAGGAG | 297 |
| RtPINK1X4R |  |  | AGCACATCAGGGTAGTCGAC |  |
| RtPARK2X6F | *PARKIN*/Ex6-9 | ENST00000366898 | TCGCAACAAATAGTCGGAACA | 298 |
| RtPARK2X9R |  |  | CCCATCTGCAGGACACACT |  |
| RtSNCAX2-3F | *SNCA*/Ex2-6 | ENST00000394986 | GTTCTCTATGTAGGCTCC | 307 |
| RtSNCAX6R |  |  | CAGGTTCGTAGTCTTGAT |  |
| RtB-ACTIN F | *ACTB*/Ex5-6 | ENST00000331789 | ATGCAGAAGGAGATCACTGC | 87 |
| RtB-ACTIN R |  |  | GATCCACACGGAGTACTTGC |  |

**S2 Table. cDNA PCR primers sequences and product sizes.**
