# Supplementary material for: Parkinson’s Disease in Saudi Patients: A Genetic Study
Source: PLoS One. 2015 Aug 14;10(8):e0135950. doi: 10.1371/journal.pone.0135950 (PMC4537238; doi:10.1371/journal.pone.0135950)
Supplement: S3 Table — (DOCX) [file pone.0135950.s010.docx]

| **GENES/NCBI-ID** | **Sample ID** | **Disease status** | **FM/SP** | **TYPE** | **CHROMSOME** | **FROM** | **TO** | **EXON / INTRON AFFECTED** | **CN STATE** | **Validation** |
| --- | --- | --- | --- | --- | --- | --- | --- | --- | --- | --- |
| *PARKIN/* NM_004562 | 19-a | affected | FM | LOSS | 6 | 162643775 | 162813995 | Exon 3/ intron 2* and 3* | 1 | No RNA |
|  | 19-b | affected sibling of 19-a | FM | LOSS | 6 | 162643775 | 162813995 | Exon 3/ intron 2* and 3* | 1 | No RNA |
|  | 21-a | affected | FM | LOSS | 6 | 162582788 | 162587775 | Intron 4 | 0 | NA |
|  | 21-a | affected | FM | LOSS | 6 | 162638345 | 162754162 | Exon 3/ intron 2* and 3* | 0 | No RNA |
|  | 21-b | unaffected son of 21-a | FM | LOSS | 6 | 162638345 | 162754162 | Exon 3/ intron 2* and 3* | 1 | No RNA |
|  | 25-a | affected | FM | LOSS | 6 | 162187069 | 162281019 | Exon 7 / intron 6* and 7* | 0 | Confirmed/Shorter transcript |
|  | 25-b | affected sibling of 25-a | FM | LOSS | 6 | 162187069 | 162280309 | Exon 7 / intron 6* and 7* | 0 | No RNA |
|  | FM-23 | no sample from family members | FM | GAIN | 6 | 162296841 | 162424430 | Exon 6/ intron 5* and 6* | 4 | No RNA |
|  | SP-103 | affected | SP | LOSS | 6 | 162618047 | 162810362 | Exon 3 and 4 / intron 2*,3 and 4* | 0 | Confirmed/PCR |
|  |  |  |  |  |  |  |  |  |  |  |

**S3 Table. Summary of the CNV analysis results.** The changes listed here are not present in database of genomic variants (DGV), but regions overlap with reported CNVs. CN state; Copy number state was scored as 0=homozygous loss, 1 heterozygous loss, 3 and above gain of copies, 2 is normal copy. * Denotes partial loss, FM; familial, SP; sporadic.
